# A recommended exercise program appropriate for patients with knee osteoarthritis: A systematic review and meta-analysis

**DOI:** 10.3389/fphys.2022.934511

**Published:** 2022-10-03

**Authors:** Xuanhui Guo, Peng Zhao, Xiao Zhou, Jialin Wang, Ruirui Wang

**Affiliations:** ^1^ College of Sports Medicine and Physical Therapy, Beijing Sport University, Beijing, China; ^2^ Sports Rehabilitation Research Center, China Institute of Sport Science, Beijing, China

**Keywords:** knee osteoarthritis, exercise program, pain, physical function, stiffness, meta-analysis

## Abstract

**Background:** Knee osteoarthritis (KOA) is a common degenerative disease. Recommended first-line management includes exercise. However, there is still no standard recommendation for the appropriate exercise program for patients with KOA.

**Purpose:** This study aims to compare the effects of a land-based exercise program with high vs. uncertain compliance with recommendations among people with KOA in pain, function, and stiffness.

**Methods:** From January 2000 to January 2022, PubMed, EBSCO, Sport-discuss, Medline, and Web of Science were searched. A comprehensive review of meta-analyses of land-based exercise programs with exercise prescriptions was done in symptomatic individuals with KOA. The Cochrane Collaboration’s standards were followed for study selection, eligibility criteria, data extraction, and statistics, and the Cochrane Collaboration’s tool was used to assess the risk of bias. Review Manager 5 software was used to extract the baseline mean and follow-up values, as well as the accompanying standard deviation, to calculate the standardized mean difference (SMD). In meta-analyses, SMD was calculated for pain outcomes, self-reported physical function, and stiffness. The effects of the outcomes on the subgroups of studies were compared. A fixed- or random-effects model was used in group research studies with comparable outcomes.

**Results:** There were 15 studies with a total of 1,436 participants. Compliance with the ACSM recommendations was categorized as “high” in five cases and “uncertain” in nine others. The SMD for pain was −0.31 (95% CI −0.47, −0.14) in the subgroup with a high ACSM compliance ratio and −0.55 (95% CI −0.69, −0.41) in the subgroup with uncertain ACSM compliance. For physical function, in the high-compliance group, the SMD was −0.21 (95% CI −0.38, −0.05), while in the uncertain-compliance group, it was −0.61 (95 % CI −0.82, −0.40). The SMD was −0.40 (95 % CI −0.61, −0.19) for stiffness and high compliance with ACSM. The SMD was −0.29 (95% CI −0.66, 0.07) for study interventions with uncertain compliance.

**Conclusion:** The results showed that the land-based training program significantly improved pain, physical function, and stiffness in KOA patients compared to controls. Exercise interventions with high adherence to ACSM recommendations differed significantly only in stiffness measures compared with the uncertain-compliance group.

**Clinical Trial Registration:** https://www.crd.york.ac.uk/prospero/#recordDetails, identifier PROSPERO (ID CRD42022311660)

## Introduction

Knee osteoarthritis (KOA) is a frequent degenerative disease characterized by joint swelling, discomfort, stiffness, functional impairment, severe muscle atrophy, and even incapacity (Vina and Kwoh, 2018; Sharma, 2021). According to the statistics, KOA impacts an estimated 302 million people globally (Collaborators, 2016), representing a 9.3% increase from 1990 to 2017 (Safiri et al., 2020). KOA affects 19% of people aged 45 years and older in the United States (Wallace et al., 2017) and roughly 18% of people in China (Bin et al., 2018). This syndrome is growing more widespread than in past decades as the population ages, and reduced levels of physical activity not only has a heavier health burden on the individual patient but also has a significant impact on the healthcare system and socioeconomic costs (Hunter et al., 2014; Prieto-Alhambra et al., 2014; Abbasi, 2017).

In this context of substantive burden, most patients with osteoarthritis do not receive appropriate management therapies (Runciman et al., 2012). Most physicians diagnose knee osteoarthritis not only by the symptoms but also by the radiological findings. The routine radiographs are read according to the Kellgren and Lawrence (K–L) classification (Kellgren and Lawrence, 1957): grade 0, no changes; grade 1, doubtful narrowing of the joint space and possible osteophytic lipping; grade 2, definite osteophytes and possible narrowing of the joint space; grade 3, moderate multiple osteophytes, substantial narrowing of the joint space and some sclerosis and possible deformity of the bone ends; grade 4, large osteophytes, marked narrowing of the joint space, severe sclerosis and definite deformity of the bone end. Conventionally, osteoarthritis has been defined as starting at K&L grade 2 or more (Kellgren and Lawrence, 1957). KOA can be classified on clinical criteria alone (including pain, age, stiffness, crepitus, bony tenderness, and bony enlargement), which make up the inclusion criteria for most clinical trials in this field (Altman et al., 1986).

Osteoarthritis is a disease that affects the entire joint, and there is no cure (Sharma, 2021). Attention should be focused on symptomatic KOA (and the phenotypes of pain and function) to identify clinically relevant investigations and treatment strategies capable of reducing the massive burden of the disease (Roos and Arden, 2016). Typical treatment management is best characterized as palliative (Hunter and Bierma-Zeinstra, 2019). Exercise is a type of physical activity that is planned, structured, and repetitive to improve or maintain one or more dimensions of physical fitness as a final or intermediate goal (Caspersen et al., 1985; Garber et al., 2011), and exercise therapy is widely used because it is easy to implement and inexpensive. Current international guidelines recommend exercise therapy as a first-line treatment for patients with KOA (Fernandes et al., 2013; Bannuru et al., 2019; Kolasinski et al., 2020).

Several meta-analyses and systematic reviews have demonstrated evidence that regular exercise of various types, including aerobic and resistance training, is effective in reducing pain and disability in those with KOA (Zhang et al., 2010; Tanaka et al., 2013; Fransen et al., 2015; Rausch Osthoff et al., 2018; Goh et al., 2019; Kraus et al., 2019). However, combined interventions of different types of exercise were excluded from these analyses. To the best of our knowledge, existing meta-analyses on exercise therapy for KOA have focused on the type, timing, and intensity of exercise (Uthman et al., 2013; Li et al., 2016; Moseng et al., 2017; Goh et al., 2019). The American College of Sports Medicine (ACSM) has created a thorough physical activity prescription for seemingly healthy individuals that includes appropriate volume and quality cardiorespiratory, resistance, flexibility, and neuromotor exercise (Garber et al., 2011). For specific recommendations, see Table 1. A review discussed that resistance training following ACSM recommendations did not differ from other training in pain and physical functional outcomes (Kraus et al., 2019). Their study only discussed the effects of recommended resistance training interventions and did not address other aspects such as flexibility and cardiorespiratory training. The benefits of exercise on pain and physical function in KOA may be due to various mechanisms. Exercise treatments must be suitably dosed to obtain physiological responses if the good effects of exercise work through physiological responses imposed by exercise (Runhaar et al., 2015). Nonetheless, there is no conventional advice for a suitable exercise dose for KOA patients, and the ideal activity program for patients with KOA is uncertain.

**TABLE 1:** American College of Sports Medicine (ACSM) recommendations for cardiorespiratory fitness, muscular strength, and flexibility in apparently healthy adults.

	Resistance exercise	Flexibility exercise	Cardiorespiratory exercise
Intensity/workload	55–90% of maximal heart rate	Stretch to the point of feeling tightness or slight discomfort	55–90% of maximal heart rate
Duration	8–12 repetitions or the number of repetitions needed to induce muscle fatigue but not exhaustion in 2–4 sets	10–30 s. 2–4 reps or 60 s total stretching time	20–90 min
Frequency	2–3 days per week	≥2–3 days per week	3–5 days per week

The purpose of this review was to investigate the impact of high or uncertain adherence to ACSM-recommended exercise regimens on pain, physical function, and stiffness in patients with KOA, specifically concerning 1) the application of the principles of ACSM recommendation in the development of the prescribed exercise within the experimental design; 2) the detailed reporting of the components of the exercise prescription in the methods, as categorized by the type of training; and 3) the collection and reporting of data on adherence of patients to the prescribed intervention. Since the optimal exercise dose for KOA patients is still unknown, we tried to explore the effects of the ACSM-recommended exercise program on KOA patients, hoping to provide a low-cost and effective exercise program for KOA patients.

## Materials and methods

### Protocol and registration

**FIGURE 1:**
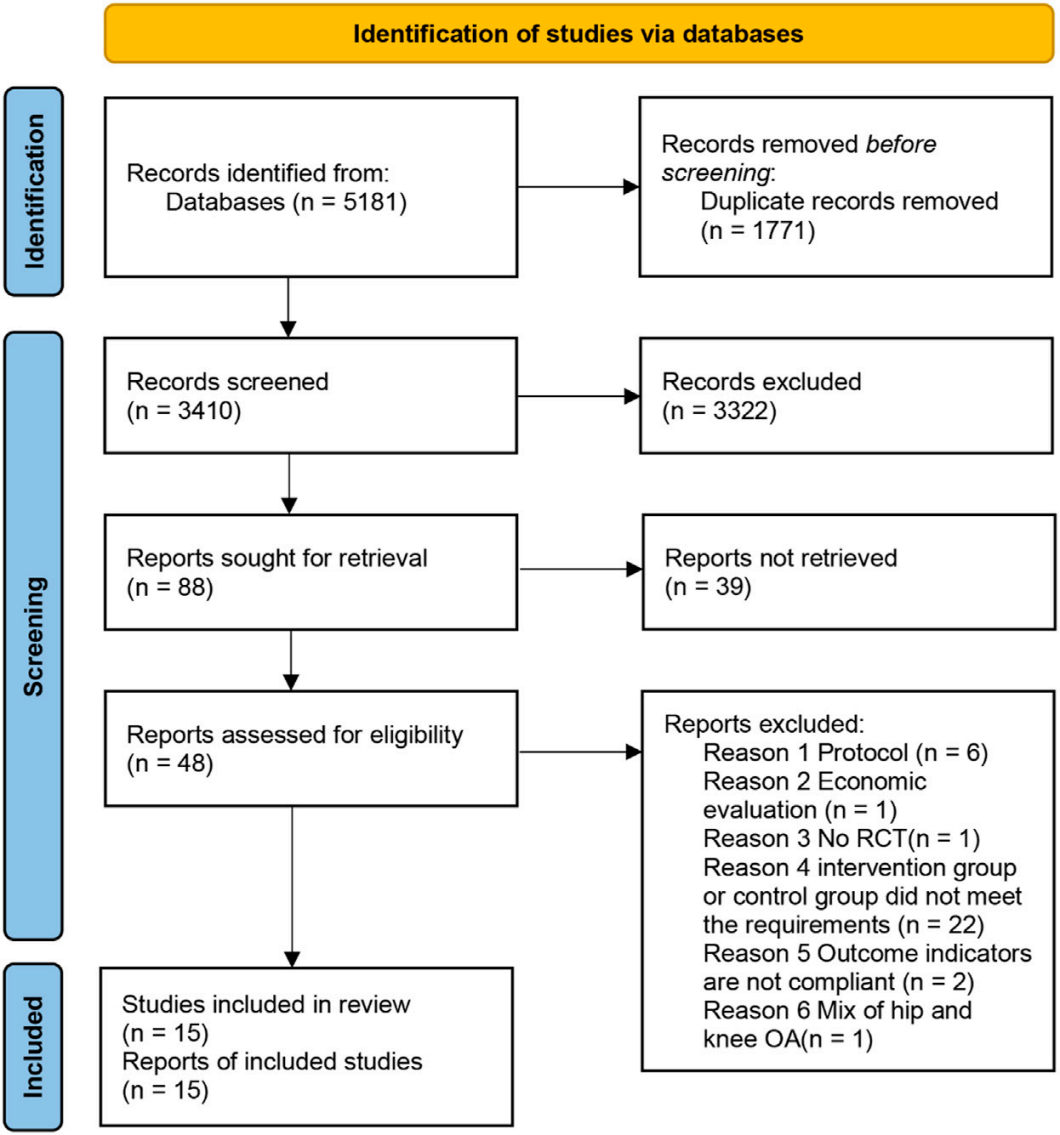
PRISMA study flow diagram.

The Preferred Reporting Items for Systematic Reviews and Meta-Analyses (PRISMA) standards were followed for conducting this meta-analysis (Page et al., 2021). Before writing, the research selection, eligibility criteria, data extraction, and standard mean difference (SMD) computations were all carried out according to a predetermined procedure and recorded in PROSPERO (ID CRD42022311660) (Booth et al., 2012). Our method is based on the PRISMA declaration and flowchart for choosing articles for review (Preferred Reporting Items for Systematic Reviews and Meta-Analyses) (Figure 1).

### Eligibility criteria

The PICOS scheme (population, intervention, control, outcome, and research design) was used to establish the study’s eligibility criteria (Riva et al., 2012). The following were the criteria for inclusion: 1) KOA patients of any age, BMI, or sex; 2) a land-based exercise program group in at least one experimental group; 3) the control group had no intervention, placebo, self-administration, or exercise intervention; 4) randomized controlled trial; and 5) only studies with patient-reported outcomes on pain, physical function, or stiffness were eligible. The following were the criteria for exclusion: 1) any physical and mental training, as well as traditional Chinese medicine training and 2) comparisons of different sports and techniques.

### Search strategy

From January 2000 to January 2022, original articles were searched in five electronic databases: PubMed, EBSCO, SPORT Discus, Medline, and Web of Science. Medical Subject Headings (MeSH) and keywords were used in the search strategy. Knee osteoarthritis, exercises, physical activity, and random randomized controlled trial were among the search phrases. The search strategy was carried out using the PICO format. For example, in PubMed, 1) for the population, the terms “Knee Osteoarthritis” OR “KOA,” 2) for the intervention, the terms “Exercises” OR “Physical Activity” OR “Activities, Physical” OR “Physical Exercise,” 3) for the outcome, the terms “Pain” OR “function” OR “physical function” OR “stiffness” OR “WOMAC.” The search strategy (Table 2) consisted of free text words and Medical Subject Headings (MeSH) terms. We also looked through the reference lists of the retrieved RCTs and earlier systematic reviews. Additional studies were found by hand-searching the bibliographies of relevant reviews and retrieved articles. When more information was requested, the research authors were contacted.

**TABLE 2:** Search strategy.

Database	Search strategy
Pubmed	#1 Osteoarthritis, knee [MeSH Terms]
	#2 Exercise [MeSH Terms]
	#3 Exercise Therapy [MeSH Terms]
	#4 pain
	#5 function
	#6 WOMAC
	#7 (#1AND#(#2 OR #3)AND(#4OR#5OR#6)) Limits: Randomized Controlled Trial or Clinical Trial
EBSCO, SPORT Discus, Medline	#1 Osteoarthritis, knee [MeSH terms]
	#2 Exercise [MeSH Terms]
	#3 Exercise therapy [MeSH terms]
	#4 Physical activity [MeSH terms]
	#5 Randomized controlled trial [MeSH Terms]
	#6 (#1 AND (#2 OR #3 OR #4)AND #5)
Web of Science	#1 Osteoarthritis, knee [MeSH Terms]
	#2 Exercise [MeSH Terms]
	#3 Exercise therapy [MeSH terms]
	#4 Physical activity [MeSH terms]
	#5 Randomized controlled trial [MeSH terms]
	#6 (#1 AND (#2 OR #3 OR #4)AND #5)

### Criteria for selection of studies

The study comprised published RCTs involving people with symptomatic KOA who had not received KOA-related surgery. Any land-based exercise program, including muscular strengthening, flexibility, and cardiorespiratory exercises, could be used as an intervention. The control group is either a blank control group or a health education group. Studies comparing distinct types of exercise programs were therefore ruled out if they failed to have a control group that did not exercise.

Two review writers (XG and XZ) independently evaluated titles and abstracts for studies that met the inclusion criteria. A complete publication text was obtained if at least one of the authors considered a study eligible. Two authors independently assessed the eligibility of the entire text. If they could not agree, a third author (JW) was chosen, and a consensus was obtained through debate. A flowchart depicts the study selection process (Figure 1). Table 5 is a list of articles that were not included in the study but were read in complete form, describing the reasons for exclusion.

### Interventions and controls

The training programs in the included studies differed significantly in terms of delivery modality, intensity, duration, repetitions, and frequency. Exercise was incorporated into all the study interventions, either alone or in combination with home exercises. The most widely used program is a comprehensive lower limb training of 8–12 repetitions per set, 3 days a week, with interventions ranging from 4 weeks to 18 months. In the meta-analysis, the impact of the recommended exercise program was explored by categorizing the intervention into two groups: high and uncertain prescription compliance according to the ACSM recommendations. A narrative method was adopted to synthesize the exercise intervention material regarding dose and adherence.

Three components were required for high compliance with ACSM-recommended intervention standards: resistance training, flexibility training, and cardiorespiratory training. The high-compliance group met three conditions, and the uncertain-compliance group included only one or two of the three. We assessed the proportion of each study’s exercise prescription that adhered to the total ACSM recommendations using this grading system. A compliance ratio of ≥60% was defined as “high compliance with ACSM guidelines,” whereas a compliance ratio of <60% was rated as “uncertain compliance with ACSM recommendations.”

The ACSM recommendations for establishing and maintaining cardiorespiratory, musculoskeletal, and neuromotor fitness in apparently healthy individuals were used to evaluate the prescribed exercise program in the included trials. Two authors (GXH and YHQ) independently assessed compliance with the suggestion by assessing each study’s exercise prescription on various criteria given for each aspect (e.g., intensity, sets, frequency, duration, and volume). Each criterion’s smallest dose was graded on a 0-2-point scale for fulfilment. Two points indicate that the standard has been met; one point demonstrates that the criterion has been met with some uncertainty, and zero points suggest that the criterion has not been met. A standard was graded as “uncertain fulfilment” if it was not reported. If the two authors (JW and RW) could not agree, a third author (HY) was chosen, and a consensus was obtained through debate.

We chose a simple blank control group that included usual care and patient education without any physical activity interventions. Usual care control was determined based on the report. In “usual care,” participants were expected to continue the routine standard of care provided by their general practitioners. Control groups that were not given any specific intervention such as “waiting list” or “usual physical activity” or where the authors did not specify the nature of the control were also classified as “usual care.”

### Outcomes

The outcomes we included in the analysis were: pain, physical function, and stiffness. The Western Ontario and McMaster Universities Osteoarthritis Index (WOMAC) scale consists of three subscales: 1) pain severity during various positions or movements, 2) severity of joint stiffness, and 3) difficulty performing daily functional activities (Collins et al., 2011). We preferred the WOMAC scale, which has been widely used internationally and is used in most trials when a study provides more than one pain and physical function assessment. The degree of stiffness is also reflected by the stiffness subscale of the WOMAC scale.

### Data synthesis and analysis

To compare the outcomes of the included studies, a meta-analysis was performed. Because the studies utilized different scales to evaluate continuous outcomes, the pooled treatment effect size was calculated using the standardized mean difference (SMD) estimation in a random-effects model.

Two reviewers (XG and RW) independently extracted data from all qualifying trials. Single session time, repetitions and sets, maximal resistance strength, frequency (number of sessions per week), total number of sessions, and overall intervention duration were recorded for the interventions (Table 4). Review Manager 5 software was used to extract the baseline mean and follow-up value, as well as the accompanying standard deviation, to calculate SMD.

If the data were incomplete or confusing, we requested additional information from relevant authors. If the two authors did not reach similar conclusions, a third author was adjudicated, and the consensus was reached through discussion. We removed studies from meta-analyses and presented the results narratively if data within a study were insufficient for pooling, even after requesting details from the authors.

The chi-square test or Higgins I^2^ values were used to measure statistical heterogeneity among studies (Huedo-Medina et al., 2006). If the I^2^ value was less than 50%, indicating low inconsistency between the results of individual trials, the findings were pooled using a fixed-effects model; otherwise, a random-effects model was used. Significance was set at *p* < 0.05.

### Quality assessment

Two reviewers (HY and LL) independently appraised the quality of studies, and the Cochrane Collaboration’s methodology was used to assess the risk of bias. Random sequence generation (selection bias), allocation concealment (performance bias), blinding of participants and personnel (performance bias), blinding of outcome assessment (detection bias), incomplete outcome data (attrition bias), selective reporting (reporting bias), and other biases were all recommended as domains to consider. If further information was needed, we asked the corresponding authors for it, and conflicts were addressed through discussion.

## Results

### Study selection

The literature search yielded 5,181 results. After duplicates were deleted, 3,410 records were screened by the title and abstract. A total of 48 articles were read in their entirety. The meta-analysis includes a total of 15 trials (16 workout groups) (Figure 1). There were six investigations with more than two study arms (Hay et al., 2006; Jan et al., 2008; Lim et al., 2008; Lin et al., 2009; Wortley et al., 2013; Braghin et al., 2018). The data from two-arm exercise only vs. waiting list control/usual care were used in these investigations.

### Study characteristics

The fifteen studies included 1,436 participants (704 comparisons and 732 interventions) with KOA. The mean age was 66 years (range 58–73), and the average proportion of female participants was 71% (range 42%–100%). Eight trials of the studies we included reported subjects’ mean K–L scores. Women were the subjects of three investigations (McCarthy et al., 2004; Foroughi et al., 2011; Borges Jorge et al., 2015).

Most included studies utilized the self-reported WOMAC scale to assess pain, physical function, and stiffness. At the same time, the Number Rating Scale (NRS) was also employed to determine outcomes. For studies with multiple follow-ups, data were extracted from immediate post-intervention assessments. Authors, year of publication, country, number of participants, age, gender, BMI, Kallgren–Lawrence (K–L) score, intervention type, and control intervention were all included in a particular standardized form developed for this review using Microsoft Excel. For details, please refer to Table 4.

Only seven trials used practical tests to evaluate a change in muscle strength, even though all fifteen included resistance training as a significant part of the intervention (Jan et al., 2008; Lim et al., 2008; Lin et al., 2009; Bennell et al., 2010; Foroughi et al., 2011; Wortley et al., 2013; Borges Jorge et al., 2015). The K–L grade was recorded in eight trials (Jan et al., 2008; Lim et al., 2008; Lin et al., 2009; Bennell et al., 2010; Foroughi et al., 2011; Oliveira et al., 2012; Wortley et al., 2013; Borges Jorge et al., 2015). Nine trials included flexibility exercises for the intervention, but none had tests to assess real flexibility change (McCarthy et al., 2004; Hay et al., 2006; Jan et al., 2008; Foroughi et al., 2011; Oliveira et al., 2012; de Matos Brunelli Braghin et al., 2019; Schlenk et al., 2020; Rafiq et al., 2021). Six studies included cardiorespiratory exercise (McCarthy et al., 2004; Hay et al., 2006; Oliveira et al., 2012; Braghin et al., 2018; de Matos Brunelli; Braghin et al., 2019; Schlenk et al., 2020), but none included any measures to evaluate a change in cardiorespiratory fitness (Table 3).

**TABLE 3:** Studies excluded with reason.

Author, year	Resistance exercise				Flexibility exercise					Cardiorespiratory exercise				Total fulfilment of ACSM criterion
		Intensity/repetition	Set	Frequency		Set	Frequency	Intensity	Time		Intensity	Duration	Frequency	(Percent)
	Contain or not	60–80% of 1RM with 8–12 reps or the number of reps needed to induce muscle fatigue but not exhaustion	2–4	2–3/d·wk	Contain or not	2–4	2–3/d·wk	Stretch to the point of feeling tightness or slight discomfort	10–30 s	Contain or not	55–90% of max HF	20–90 min	3–5/d·wk	
Braghin et al. (2018)	Contain	30%–70% 1RM, advance gradually, 10 reps	3	2	Contain	NR	NR	5 min	2	Contain	65–70% MHR, 85%–90%	20 min	2	70
Braghin, (2019)	Contain	30%–70% 1RM, advance gradually, 10 reps	3	2	Contain	NR	NR	5 min	2	Contain	65–70% MHR, 85%–90%	20 min	2	70
McCarthy et al. (2004)	Contain	6 reps	4	2 + home exercise	Contain	4	2 + home exercise	NR	2	Contain	NR	20 min	2	80
Oliveira et al. (2012)	Contain	50%–60% 1RM, 15 reps	3	2	Contain	NR	30 s	3 reps	2	Contain	NR	10 min	2	80
Rafiq et al. (2021)	Contain	2 levels of difficulty, 7 or 10 reps	3	3	Contain	NR	NR	10 reps	3	3				60
Schlenk et al. (2020)	Contain	low to moderate	3	2–3	Contain	NR	NR	NR	2–3	Contain	NR	Total 150 min	2–3	60
Bennell et al. (2010)	Contain	10 reps	3	5	Not					Not				30
Borges Jorge et al. (2015)	Contain	50%–70% 1RM, 8 reps	2	2	Not					Not				30
		Intensity/repetition	Set	Frequency		Set	Frequency	Intensity	Time		Intensity	Duration	Frequency	(Percent)
	Contain or not	60–80% of 1RM with 8–12 reps or the number of reps needed to induce muscle fatigue but not exhaustion	2–4	2–3/d·wk	Contain or not	2–4	2–3/d·wk	Stretch to the point of feeling tightness or slight discomfort	10–30 s	Contain or not	55–90% of max HF	20–90 min	3–5/d·wk	
Foroughi et al. (2011)	Contain	3 reps	3	3	Contain	NR	NR	NR	3	Not				40
Hay et al. (2006)	Contain	Ind. tail	Ind. tail	Home exercise	Contain	NR	NR	NR	NR	Contain	NR	NR	NR	30
Jan et al. (2008)	Contain	high-resistance exercise	3	3	Contain	10 min	NR	NR	3					50
Lim et al. (2008)	Contain	10 reps	2–3	Home exercise 5 days	Not					Not				30
Lin et al. (2009)	Contain	50%–70% of 1RM, 6 reps	4	3	Not					Not				30
Nelligan et al. (2021)	Contain	3 levels of difficulty, 10 reps	3	3 + home exercise	Not					Not				30
Wortley et al. (2013)	Contain	8–12 reps	2–3	2	Not					Not				30

ACSM, American College of Sports Medicine; Ind. tail, individually tailored; and NR, not reported.

**TABLE 4:** Exercise interventions evaluated according to the American College of Sports Medicine's (ACSM) recommendations.

Author, year	Country	Intervention group	Control group	Intervention and control	Outcome (pain)	Outcome (function)	Outcome (stiffness)	Applied intervention and relevant measure		
		n = Age% female:BMIKL	n = Age% female:BMIKL					Resistance	Flexibility	Cardiorespiratory
Braghin et al. (2018)Brazil	Brazil	15	16	8 weeks of physical exercises, 2 days a week vs. control (did not perform the exercise program)	WOMAC pain subscale	WOMAC function subscale		No outcome measure	No outcome measure	No outcome measure
		59.42 ± 8.06	60.19 ± 9.28							
		66.7%	87.5%							
		30.21 ± 4.63	31.10 ± 6.96							
		NR	NR							
Braghin, (2019)	Brazil	15	15	8 weeks of physical exercises 2 days a week vs. control (low-level laser)	WOMAC pain subscale	WOMAC function subscale		No outcome measure	No outcome measure	No outcome measure
		58.57 ± 7.42	60.8 ± 9.2							
		66.7%	80%							
		29.28 ± 4.72	31.52 ± 6.97							
		NR	NR							
McCarthy et al. (2004)	United Kingdom	104	86	8 weeks of class-based exercise program twice a week in addition to home-based exercise program vs. control	WOMAC pain subscale	WOMAC function subscale	WOMAC stiffness subscale	No outcome measure	No outcome measure	No outcome measure
		100%	100%							
		64.0 ± 9.7	64.5 ± 9.9							
		30.2 ± 5.3	29.4 ± 5.2							
		NR	NR							
Oliveira et al. (2012)	Brazil	50	50	8 weeks of stationary bicycle, hamstrings stretching, and quadriceps strengthening 2 days a week vs. control	WOMAC pain subscale	WOMAC function subscale	WOMAC stiffness subscale	No outcome measure	No outcome measure	No outcome measure
		61.50 ± 6.94	58.78 ± 9.60							
		94%	90%							
		30.00 ± 5.05	29.72 ± 4.11							
		2	2							
Rafiq et al. (2021)	Malaysia	25	25	4 weeks of lower limb rehabilitation protocol (LLRP) 3 days a week vs. control	WOMAC pain subscale	WOMAC function subscale	WOMAC stiffness subscale	No outcome measure	No outcome measure	No outcome measure
		53.40 ±1 5.18	52.84 ± 5.74							
		56%	52%							
		32.18 ± 4.49	32.01 ± 3.89							
		NR	NR							
Schlenk et al. (2020)	United States	91	91	72 weeks of lower extremity exercise (LEE) 2–3 days a week vs. attention-control	WOMAC pain subscale	WOMAC function subscale		No outcome measure	No outcome measure	No outcome measure
		64.47 ± 8.46	64.96 ± 7.76							
		73.6%	72.5%							
		34.5 ± 7.48	33.64 ± 6.43							
		NR	NR							
Bennell et al. (2010)	Australia	39	37	12 weeks supervised home-based exercise program targeting the hip abductor and adductor muscles vs. control	WOMAC pain subscale	WOMAC function subscale		Hip abduction and adductor strength	No outcome measure	No outcome measure
		64.5 ± 9.1	64.6 ± 7.6							
		48.9%	54.5%							
		27.5 ± 4.7	28.4 ± 4.1							
		3	3							
Borges Jorge et al. (2015)	Brazil	29	29	12 weeks progressive resistance exercise program 2 days a week vs. control	WOMAC pain subscale	WOMAC function subscale	WOMAC stiffness subscale	one-repetition maximum (1RM)	No outcome measure	6 MW
		61.7 ± 6.4	59.9 ± 7.5							
		100%	100%							
		30.6 ± 5.75	31.4 ± 4.42							
		2	1							
Foroughi et al. (2011)	Australia	26	28	6-month high-intensity (80%) resistance exercise program vs. sham exercise program (minimal-resistance during exercise)	WOMAC pain subscale	WOMAC function subscale	WOMAC stiffness subscale	First peak knee adduction moment and hip adduction moment	No outcome measure	No outcome measure
		66 ± 8	65 ± 7							
		100%	100%							
		31.4 ± 5.4	32.7 ± 8.4							
		3	3							
Hay et al. (2006)	United Kingdom	108	108	12 weeks of community physiotherapy including general aerobic exercise and specific muscle strengthening exercises (non-weight bearing and weight bearing) and stretching exercise vs. control	WOMAC pain subscale	WOMAC function subscale		No outcome measure	No outcome measure	No outcome measure
		67.9 ± 8.5	68.2 ± 8.0							
		65%	65%							
		Healthy 25%, overweight 49%	Healthy 19%, overweight 41%							
		Obese 26%	Obese 41%							
		NR	NR							
Jan et al. (2008)	Taiwan, China	34	30	8 weeks of high-resistance exercise (60% of 1RM) vs. control	WOMAC pain subscale	WOMAC function subscale		Measurement of knee extensor and flexor torque	No outcome measure	No outcome measure
		63.3 ± 6.6	62.8 ± 6.3							
		79.4%	83%							
		NR	NR							
		3	3							
Lim et al. (2008) More neutrally aligned	Australia	26	26	12 weeks of quadriceps strengthening 5 days a week vs. control	WOMAC pain subscale	WOMAC function subscale		Quadriceps strength	No outcome measure	No outcome measure
		67.2 ± 6.7	66.6 ± 8.9							
		50%	46%							
		28.2 ± 3.7	30.3 ± 5.3							
		3	3							
Lim et al. (2008) More misaligned	Australia	27	28	12 weeks of quadriceps strengthening 5 days a week vs. control	WOMAC pain subscale	WOMAC function subscale		Quadriceps strength	No outcome measure	No outcome measure
		64.1 ± 9.3	60.8 ± 7.8							
		63%	61%							
		29.0 ± 5.2	28.4 ± 5.0							
		3	3							
Lin et al. (2009)	Taiwan, China	36	36	8 weeks of non–weight-bearing exercises. 3 days a week vs. control	WOMAC pain subscale	WOMAC function subscale		Knee Extensors and Flexors Strength	No outcome measure	No outcome measure
		61.6 ± 7.2	62.2 ± 6.7							
		66.6%	72.2%							
		NR	NR							
		3	3							
Nelligan et al. (2021)	Australia	103	103	24 weeks of Web-based strengthening exercise and physical activity program 3 days a week vs. control	NRS	WOMAC function subscale		No outcome measure	No outcome measure	No outcome measure
		60.3 ± 8.2	59.0 ± 8.5							
		58%	64%							
		31.1 (26.6–34.9)	31.6 (26.9–36.4)							
		NR	NR							
Wortley et al. (2013)	United States	104	86	8 weeks of class-based exercise program twice a week in addition to home-based exercise program vs. control	WOMAC pain subscale	WOMAC function subscale	WOMAC stiffness subscale	No outcome measure	No outcome measure	No outcome measure
		100%	100%							
		64.0 ± 9.7	64.5 ± 9.9							
		30.2 ± 5.3	29.4 ± 5.2							
		NR	NR							
		100%	100%							
KL, Kellgren–Lawrence (K–L) score; NR, not reported										

**TABLE 5:** Study characteristics.

Studies excluded with reason.	
Study	Reason for exclusion
Adhama et al. (2021)	Protocol for a randomized controlled trial
Veenhof et al. (2006)	No exercise intervention
Alfieri et al. (2020)	Manual in control intervention
de Paula Gomes et al. (2020)	Exercise in control intervention
León-Ballesteros et al. (2020)	Manual in control intervention
Chao et al. (2021)	Medication in the control group
Moreira et al. (2021)	Exercise in control intervention
Nigam et al. (2021)	Manual in control intervention
Nejati et al. (2015)	Acupuncture combined with exercise intervention
Reza et al. (2021)	Manual in control intervention
Knoop et al. (2013)	Exercise in control intervention
Bennell et al. (2014)	Exercise in control intervention
Øiestad et al. (2013)	Protocol for a randomized controlled trial
Lange et al. (2009)	Protocol for a randomized controlled trial
Coupé et al. (2007)	Economic evaluation
Mangani et al. (2006)	Comorbidity index
Heuts et al. (2005)	Mix of hip and knee OA
Diracoglu et al. (2005)	Exercise in control intervention
Deyle et al. (2005)	Manual in control intervention
Ojoawo et al. (2016)	Exercise in control intervention
Kabiri et al. (2018)	Exercise in control intervention
Keogh et al. (2018)	Exercise in control intervention
Alkhawajah and Alshami, (2019)	Manual in control intervention
Allen et al. (2019)	Protocol for a randomized controlled trial
Nazari et al. (2019)	Exercise in control intervention
Alfredo et al. (2020)	Exercise in control intervention
Yilmaz et al. (2019)	Exercise in control intervention
Holm et al. (2020)	Exercise in control intervention
Knoop et al. (2020)	Protocol for a randomized controlled trial
Cuyul-Vásquez and Fuentes, (2021)	Protocol for a randomized controlled trial
Onwunzo et al. (2021)	Outcome indicators are not compliant
Anwer and Alghadir, (2014)	Outcome indicators are not compliant
Chen et al. (2019)	No RCT

### Risk of bias

**FIGURE 2:**
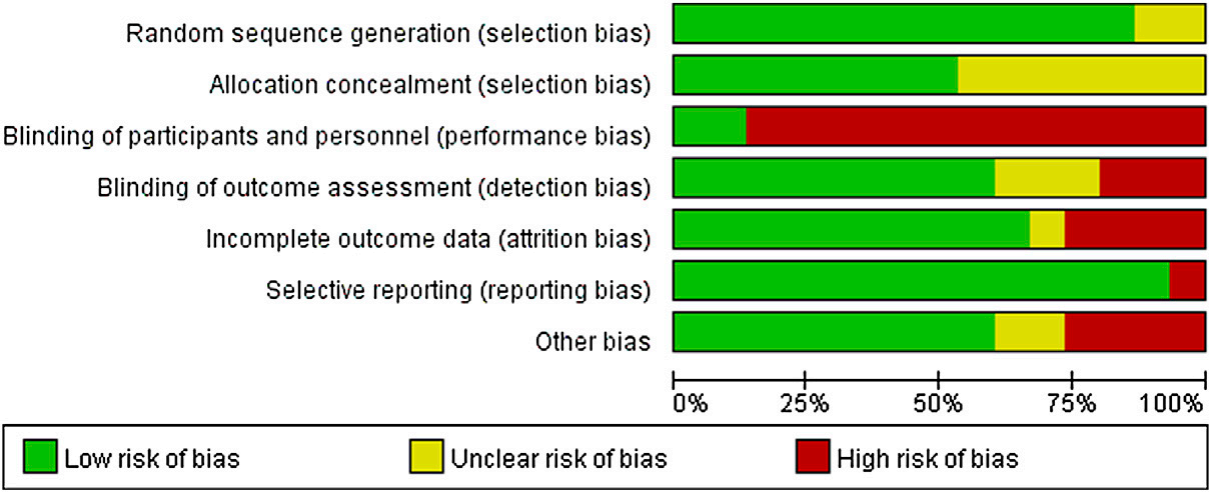
Combined percentage risk of bias in each risk domain for all included trials.

**FIGURE 3:**
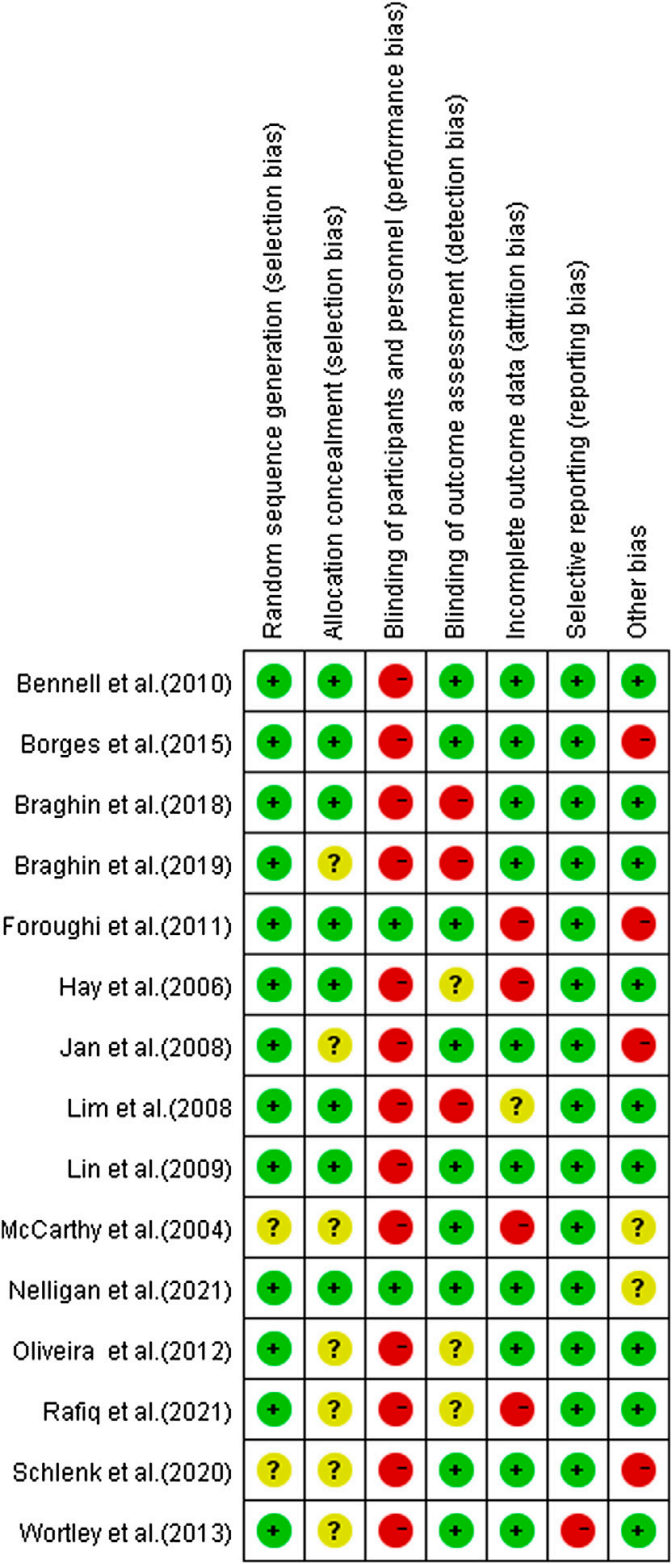
Risk of bias summaries for all exercise trials.

For random sequence generation, all studies were deemed to have a low risk of bias (Figure 2 and Figure 3). Except for one trial that used pseudorandom (Wortley et al., 2013) and the other simple randomization (Schlenk et al., 2020), all the included RCTs used a computer or random table to produce random sequences. The lack of double blinding was the most common source of possible methodological bias. Only two of the 15 studies included in this review stated that participants and result assessors were blinded (Foroughi et al., 2011; Nelligan et al., 2021). Nine investigations showed a minimal risk of blinding (McCarthy et al., 2004; Jan et al., 2008; Lin et al., 2009; Bennell et al., 2010; Foroughi et al., 2011; Wortley et al., 2013; Borges Jorge et al., 2015; Schlenk et al., 2020; Nelligan et al., 2021). For the item intent-to-treat, four studies presented a high risk (McCarthy et al., 2004; Hay et al., 2006; Foroughi et al., 2011; Rafiq et al., 2021).

### Compliance with the ACSM recommendations

As many trials did not adequately describe the treatment, the authors used a specialized scoring system to judge adherence to ACSM. The intervention groups included in the trials were categorized based on the dose of exercise into two groups: high compliance and uncertain compliance. For six studies (McCarthy et al., 2004; Oliveira et al., 2012; de Matos Brunelli Braghin et al., 2019; Schlenk et al., 2020; Rafiq et al., 2021), the exercise interventions had a compliance ratio of ≥60% with the ACSM recommendations, while for nine studies (Hay et al., 2006; Jan et al., 2008; Lim et al., 2008; Lin et al., 2009; Bennell et al., 2010; Foroughi et al., 2011; Wortley et al., 2013; Borges Jorge et al., 2015; Braghin et al., 2018; Nelligan et al., 2021),the exercise interventions had a compliance ratio of <60% (Table 4). There are two main reasons for the study interventions with a compliance ratio of <60%. One was that the experimental design did not address all aspects of the recommended prescription; the other was that adequate exercise prescribing information was not provided to allow for a proper assessment.

### Meta-Analyses

**FIGURE 4:**
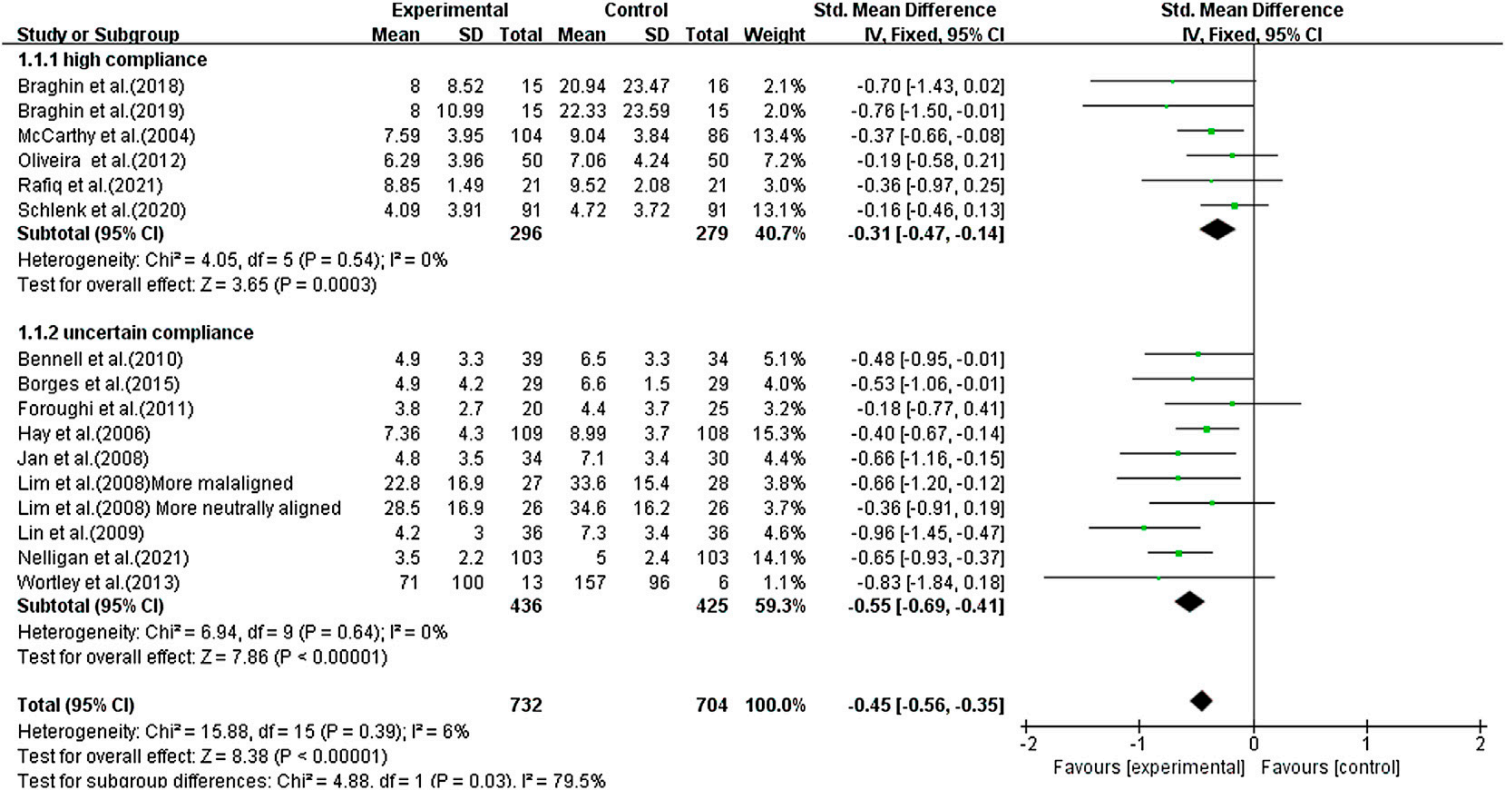
Forest plot of comparison: high versus uncertain, outcome: 1.1 Pain. Effect of land-based exercise compared to control when pain outcomes were assessed using the WOMAC pain subscale or NRS. Data are presented as standardized mean differences, with differences <0 favoring land-based exercise. Subgroup analysis showed similar effects for pain in the high-compliance group [SMD −0.31 (95% CI −0.47, −0.14), I^2^ = 0%] (*p* < 0.001) and those with the uncertain-compliance group [SMD −0.55 (95% CI −0.69, −0.41), I^2^ = 0%] (*p* < 0.001).

**FIGURE 5:**
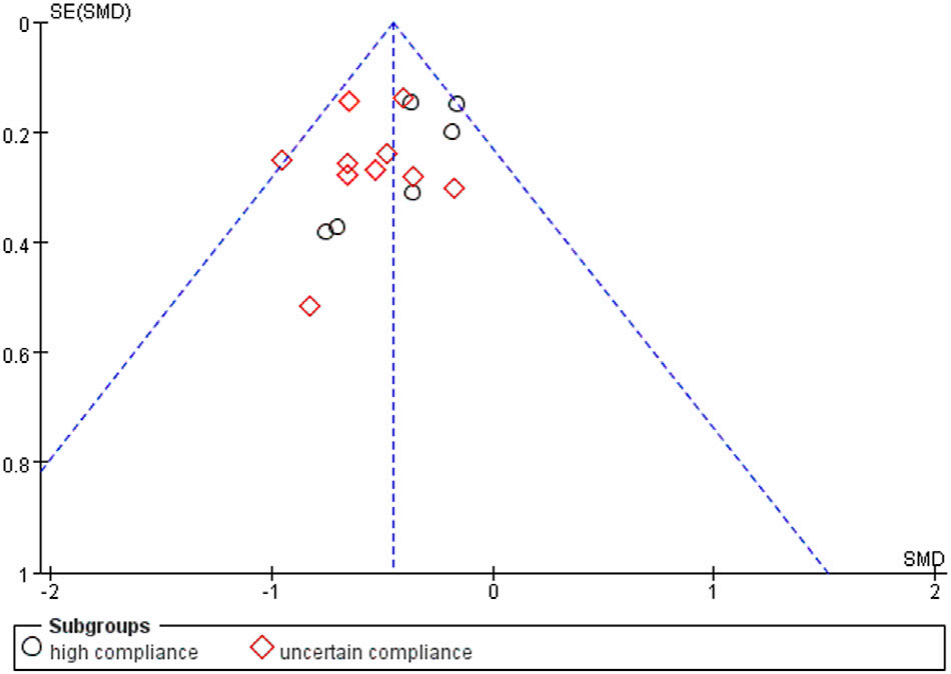
Funnel plots for pain: Symmetric funnel plots showed no publication bias. SE, standard error; SMD, standardized mean difference.

Using a fixed-effects model, the overall pooled SMD for pain was moderate with −0.45 (95% CI −0.56, −0.34) favoring activity over no exercise (Figure 4). The SMD was −0.31 (95% CI −0.47, −0.14) (*p* < 0.001) for the subgroup with a high-ACSM compliance ratio. The SMD for the subgroup with uncertain ACSM compliance was −0.55 (95% CI −0.69, −0.41) (*p* < 0.001), and there was no heterogeneity (0%) among the various trials for the outcome measure pain in the high and uncertain subgroups. Symmetry was visible in the funnel plot (Figure 5), and the Egger test showed *p* = 0.155, indicating no publication bias.

**FIGURE 6:**
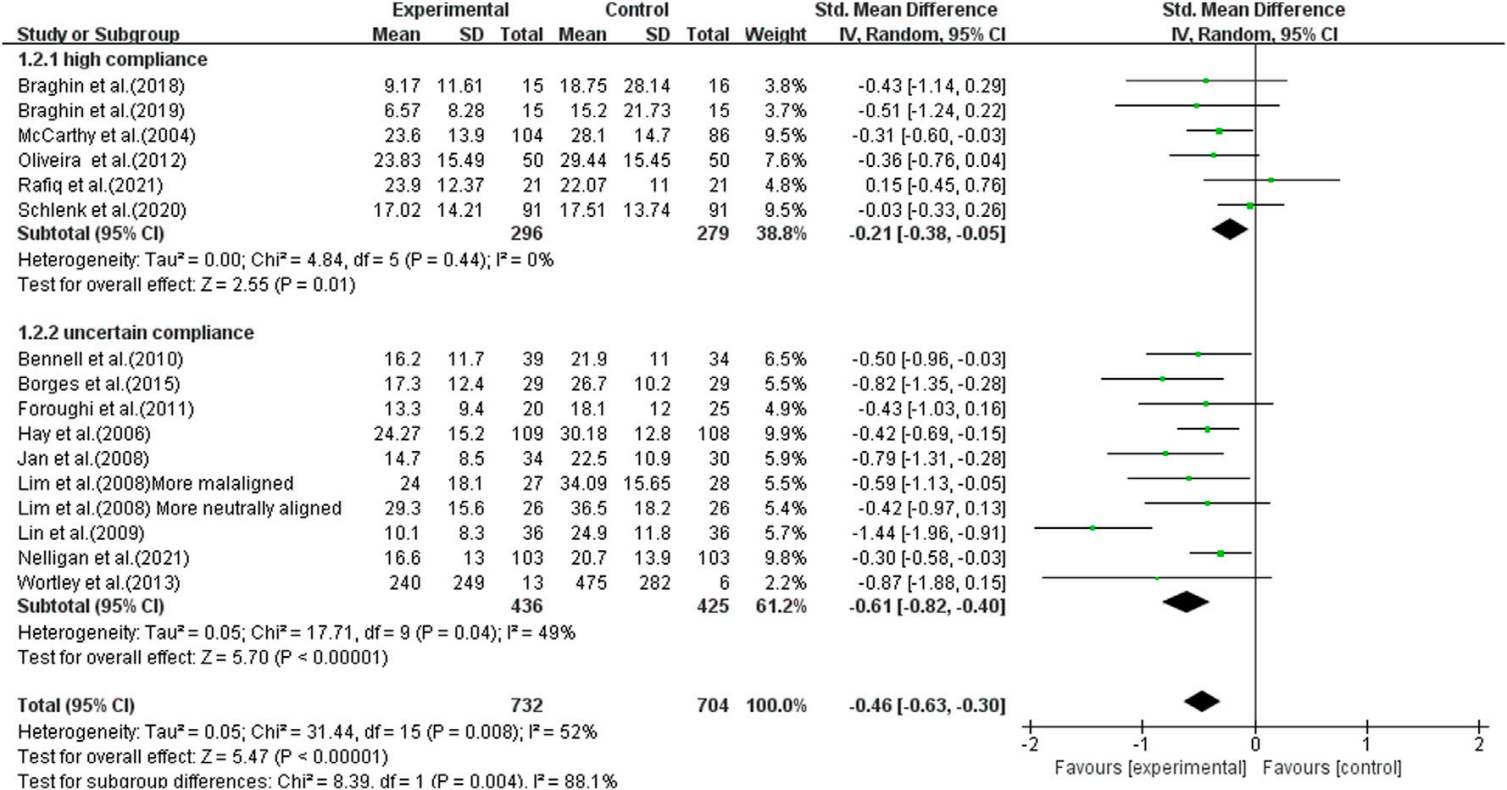
Forest plot of comparison: high versus uncertain, outcome: 1.2 Physical function (WOMAC). A random-effects model was used because of significant heterogeneity (l^2^ = 52%). Effect of land-based exercise compared to control when physical function outcomes were assessed using the WOMAC function subscale. Data are presented as standardized mean differences, with differences <0 favoring land-based exercise. Subgroup analysis showed similar effects for physical function in the high-compliance group [SMD −0.21 (95% CI −0.38, −0.05), I^2^ = 0%] (*p* = 0.010) and those with the uncertain-compliance group [SMD −0.61 (95% CI −0.82, −0.40), I^2^ = 49%] (*p* < 0.001).

**FIGURE 7:**
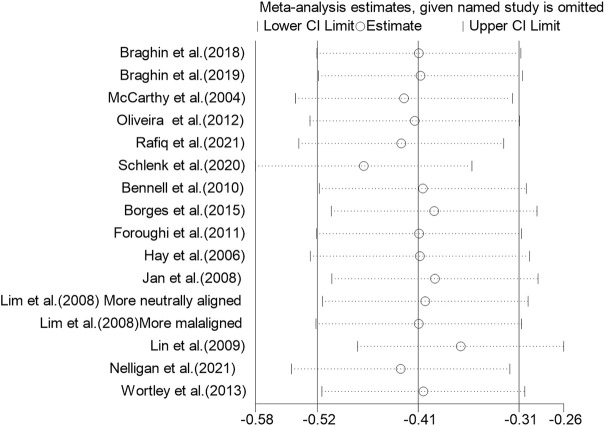
Sensitivity analysis plot: The literature data were eliminated one by one, and the results showed that no single article had a greater impact on the overall.

**FIGURE 8:**
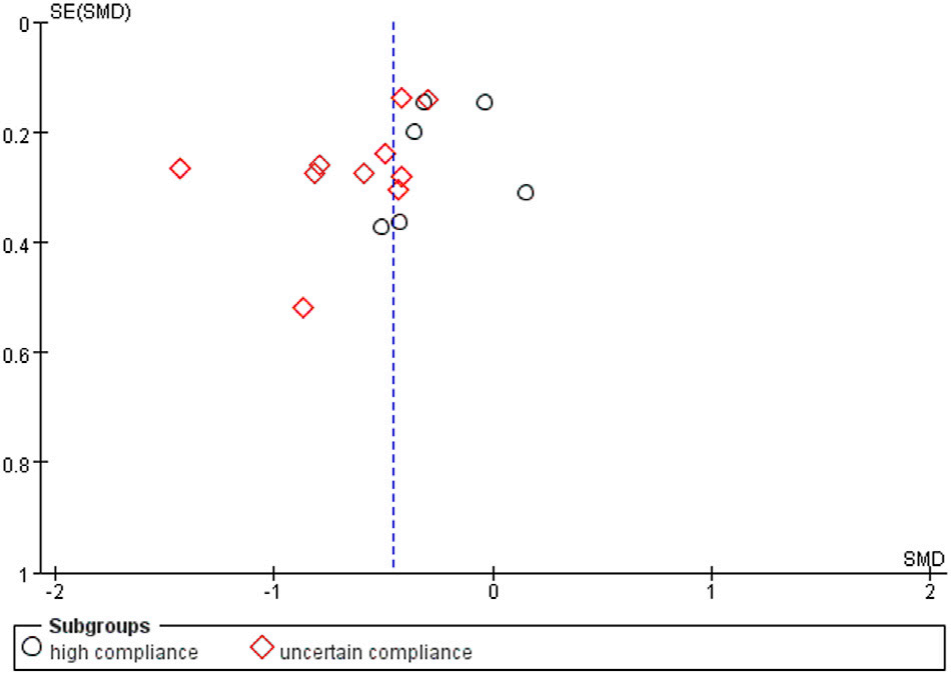
Funnel plots for physical function: Symmetric funnel plots showed no publication bias. SE, standard error; SMD, standardized mean difference.

Using a random-effects model, the overall pooled SMD for physical function was −0.43 (95% CI −0.68, −0.41), favoring exercise over no exercise (Figure 6). The pooled SMD for the study interventions with high ACSM compliance was −0.21 (95 % CI −0.38, −0.05) (*p* = 0.010) in the subgroup analysis. The SMD was −0.61 (95% CI −0.82, −0.40) (*p* < 0.001) for study interventions with ambiguous compliance. For physical function, the heterogeneity between individual studies was low (0%) for the grouping with high compliance and moderated to large (49%) for the subgroup with uncertain compliance. To investigate sources of heterogeneity, we employ sensitivity analysis. Our data are robust, according to a sensitivity analysis (Figure 7). Symmetry was visible in the funnel plot (Figure 8), and the Egger test showed *p* = 0.092, indicating no publication bias.

**FIGURE 9:**
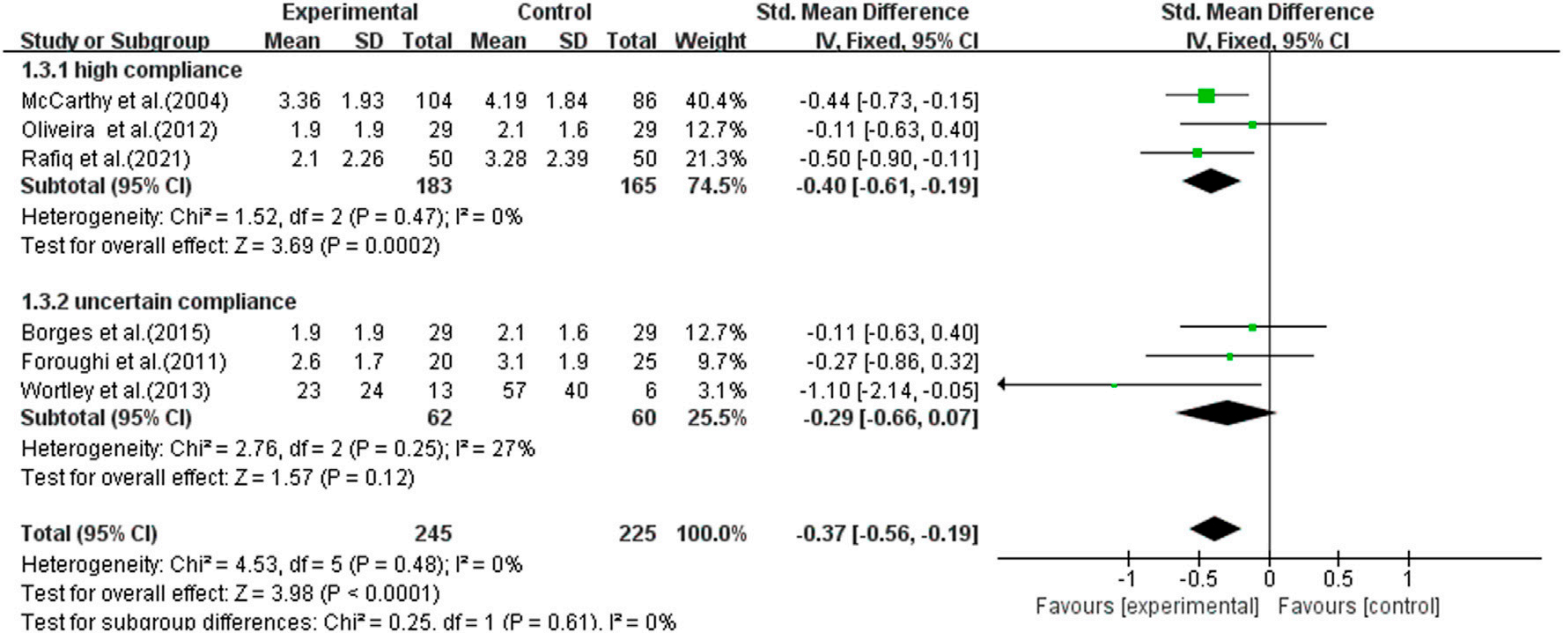
Forest plot of comparison: high versus uncertain, outcome: 1.3 Stiffness (WOMAC). Effect of land-based exercise compared to control when stiffness outcomes were assessed using the WOMAC stiffness subscale. Data are presented as standardized mean differences, with differences <0 favoring land-based exercise. Subgroup analysis showed that the high-compliance group [SMD −0.40 (95% CI −0.61, −0.19), I^2^ = 0%] (*p* < 0.001) showed significant improvement in stiffness measures and the uncertain-compliance group [SMD −0.29 (95% CI −0.66, 0.07), I^2^ = 27%] (*p* = 0.120) did not. Subgroup analysis showed similar effects for pain in the high-compliance group [SMD −0.21 (95% CI −0.38, −0.05), I^2^ = 0%] (*p* = 0.010) and those with the uncertain-compliance group [SMD −0.61 (95% CI −0.82, −0.40), I^2^ = 49%] (*p* < 0.001).

**FIGURE 10:**
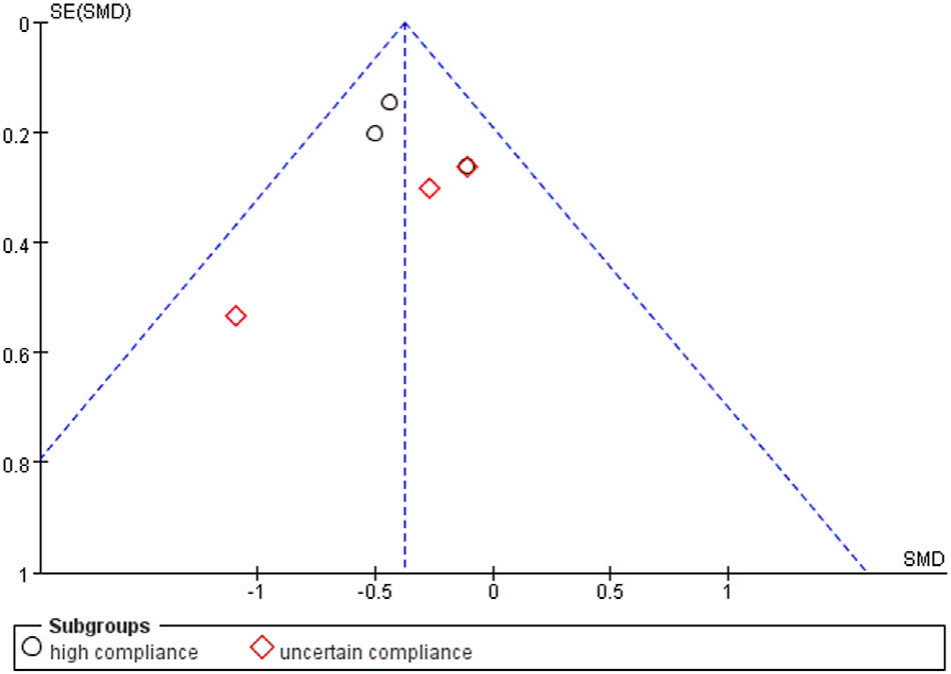
Funnel plots for stiffness; symmetric funnel plots showed no publication bias. SE, standard error; SMD, standardized mean difference.

The overall pooled SMD for stiffness was −0.37 (95% CI −0.56, −0.19), favoring exercise over no exercise (Figure 9). The SMD was −0.40 (95% CI −0.61, −0.19) (*p* < 0.001) in the subgroup analysis for study interventions with high ACSM compliance. The SMD was −0.29 (95% CI −0.66, 0.07) (*p* = 0.120) for study interventions with uncertain compliance. For the category with uncertain compliance, the heterogeneity among the separate studies for pain was negligible (27%), implying no publication bias. Symmetry is depicted in a funnel plot (Figure 10), and the Egger test showed *p* = 0.861, indicating no publication bias.

## Discussion

This systematic review and meta-analysis confirmed that a supervised land exercise regimen was superior to controls in pain, function, and stiffness in KOA patients. After calculating the compliance score, a subgroup analysis was performed after comparing it to the ACSM-recommended exercise schedule. There was no statistically significant improvement in pain or physical function in the high-compliance group (≥60% compliance with ACSM recommendations) compared with the uncertain-compliance group (<60% compliance with ACSM recommendations). However, on the stiffness metric, the high-compliance subgroup outperformed the uncertain-compliance group. In contrast to previous meta-analyses (Tanaka et al., 2013) that ignored the effects of combined exercise interventions, the focus of this article is that since resistance, cardio, and flexibility are the most common training modalities, studies of multidimensional exercise interventions can provide a broader range of program recommendations for future studies, rather than being limited to one specific exercise type study.

Our finding is that the high-compliance group significantly improved stiffness indicators. Stiffness may reflect muscle changes per se (Reid and McNair, 2010). A previous meta-analysis (Li et al., 2016) of resistance training showed no significant therapeutic benefit in stiffness for both high- and low-intensity resistance training. One explanation for the disadvantage of mixing exercise with resistance training and aerobic exercise in the same session may be the molecular response, where resistance training increases myofibrillar protein response, and aerobic exercise increases mitochondrial content in the muscle. This molecular response decreases when aerobic and resistance exercises are performed simultaneously (Hawley, 2009). Based on this, we speculate that the better performance of the high-compliance group may be due to the inclusion of flexibility training in the mixed intervention program of the high-compliance group. Flexibility training has been shown to benefit KOA patients (Reid and McNair, 2010; Suzuki et al., 2019). KOA may lead to changes in muscle activation patterns, such as decreased quadriceps activity used to accommodate pain and act on the knee. Increased hamstring activity may also lead to failure to fully extend and increased stiffness (Trudel and Uhthoff, 2000). There is evidence that flexibility training can improve hamstring tension and reduce stiffness in KOA patients (Reid and McNair, 2010).

A previous network meta-analysis by Goh et al. (2019) proved that exercise treatment programs focusing on a single form of exercise were more effective in reducing pain and patient-reported disability than those combining multiple types of exercise with distinct goals in the same session. Our findings support their conclusions on pain and physical function measures. We found that there was no statistically significant improvement in pain and functional measures between the high-adherence group and the uncertain-adherence group (pain: high SMD = −0.31, 95% CI −0.47 to −0.14; uncertain SMD = −0.55, 95% CI −0.69, to −0.41 and physical function: high SMD = −0.21, 95 % CI −0.38 to −0.05; uncertain SMD = −0.61, 95% CI −0.82 to −0.40). Attendance and engagement of KOA patients also deserve consideration by researchers, such as the intensity of the individual components was insufficient or poorly adhered to due to the complexity of the regimen compared with a single exercise program. Particularly when considering that there are many domains of physical impairment in people with KOA, it may be that the lack of response to mixed exercise reflects the flawed implementation of the program (Goh et al., 2019).

In eight study trials, we included reported subjects’ mean K–L scores, and none exceeded grade III. Managing pain may be the primary concern for patients with K–L grade IV. KOA has several clinical subgroups, including medial and lateral tibiofemoral osteoarthritis, patellofemoral KOA, and others. Current research studies on KOA typing treatments are conflicting, and it is uncertain which patients will benefit from which therapy (Deveza et al., 2019). Only one trial in our study (Lim et al., 2008) classified patients with different knee arrangements and controls. This experiment investigated whether quadriceps strengthening for 12 weeks in patients with medial KOA had different effects on knee adduction torque, pain, and function than in patients with and without varus deformity. Their findings were that quadriceps strengthening did not significantly affect knee adduction torque in participants with more varus or neutral alignment. They suggest that future studies evaluating the efficacy of interventions should stratify analyses by local factors. The extreme interpatient heterogeneity in clinical and anatomical symptoms of KOA is a significant feature of the disease (Felson, 2010; Bierma-Zeinstra and Verhagen, 2011). One of the reasons why a uniform treatment approach for patients with KOA is impossible is because of this diversity.

Most of the exercise interventions included in this meta-analysis involved training of the entire lower extremity, with only one experiment (Lim et al., 2008) discussing the impact of quadriceps. The lower extremities have formed a whole kinematic chain, making it impossible for the hip, knee, or ankle joints to work entirely independently. Instead, they may affect each other. For example, hip abductor strengthening exercises can reduce pain and improve overall function in people with KOA (Sled et al., 2010; Xie et al., 2018). The balance of strength between the quadriceps and hamstrings is critical for reducing the risk of KOA (Orssatto et al., 2018; Muollo et al., 2022). The latest research study also shows that the performance of the gastrocnemius and Achilles tendon is also altered in KOA patients (Chen et al., 2021). While these studies show the effect of different muscle groups on KOA patients, the application of training principles in exercise design is inconsistent and underreported, and a proper scientific evaluation of the literature is difficult.

## Study strengths and limitations

The strength of this review is the solid research methodology. First, to our knowledge, this is the first meta-analysis of KOA exercise therapy grouped by ACSM adherence. Our main objective was to explore the effectiveness of a comprehensive multidimensional exercise program for KOA patients Subgroup analysis is particularly relevant to this, given that exercise is a complex and multifaceted intervention, and exercise intervention programs are challenging to quantify. We realize that this approach does not allow analysis of more specific or detailed components of exercise interventions investigated in a small number of trials, such as tai chi, and they cannot be classified according to exercise volume. Therefore, additional body–mind exercises and traditional Chinese medicine exercise categories are not included (such as undetermined doses of tai chi, yoga, and meditation). This allows us to focus more on the dose of the land-based training program. Second, we gathered data from 2000 to 2022, excluding older studies that may have lacked a thorough description of the exercise intervention and dosage. Third, “usual care” is not standardized and varies considerably between studies (Goh et al., 2019). Many published reports in osteoarthritis extend controls to include other non-exercise interventions (e.g., patient education and behavioral therapy) rather than limiting them to “usual care” (Tanaka et al., 2013; Fransen et al., 2015). So, the study’s firm basis is that the control group is a blank control group that receives no exercise intervention and excludes those who receive exercise education as part of their health education.

There are several limitations to our study. One significant flaw is that we entirely relied on author descriptions to classify high and doubtful compliance. Even when the exercise focuses on strength improvement, it is typical to find some elements of flexibility and aerobic training in the program. Some trials did not fully characterize exercise doses, describing them merely as “individualised” in some cases (Hay et al., 2006; Foroughi et al., 2011; Schlenk et al., 2020). Due to a lack of experimental description, the study intervention could be incorrectly classed as an uncertain-compliance group using such a score method. The exercise intervention regimen in future KOA trials should be more precise, allowing researchers to continue investigating the probable dosage response. The second limitation is that blinding is exceedingly difficult or even impossible. In studies of exercise vs. no exercise, there is a risk that the treatment effect sizes may be inflated. A final limitation is that our analysis was not according to ITT (intention-to-treat) because some studies did not report ITT data, which may bias our results.

## Implications for practice/research

Even though thereare some limitations to this review, there are also some implications for practice. Exercise programs combining strengthening exercise with exercise aimed at increasing flexibility and aerobic capacity seem to be the option healthcare providers can offer patients. The Osteoarthritis Research and Society International (OARSI) recommended that osteoarthritis patients should be encouraged to undertake regular aerobic, muscle strengthening, and range of motion exercises (Zhang et al., 2008; Zhang et al., 2010). Optimal exercise programs for KOA should have one goal and focus on combining strengthening with flexibility and aerobic exercises, according to a previous meta-analysis (Uthman et al., 2013), investigating the effect of lower limb exercise on KOA pain and disability. These elements are currently at the center of the creation of land-based training regimens for KOA. The results of our study provide evidence to support this recommendation.

None of the experiments achieved a perfect score according to our scoring system. We discovered in our meta-analysis that whereas resistance training details seem to be precise descriptions, flexibility and cardiorespiratory training information are scant, with only two studies by the same author addressing each aspect of the ACSM intervention (Braghin et al., 2018; de Matos Brunelli Braghin et al., 2019). Future KOA studies should include more multidimensional experiments, such as the combination of resistance training, flexibility training, and cardiorespiratory training. The design of the intervention should reflect these goals, including the correct selection of the type of exercise and the detailed address of the training principles (specificity, overload, and progression). The appropriate profile of repetitions, sets, intensity, rest, and progression will be achieved. The treatment of patients with KOA should indeed be individualized, but the premise of such individualization is that there are specific classification standards and recommended ranges. We recommend that future investigators conduct further research in patients with KOA to explore whether the location and degree of KOA influence the effect of exercise interventions.

## Conclusion

The meta-analysis showed improvements in pain, physical function, and stiffness in the high-compliance group (≥60% compliance with ACSM recommendations) compared with the control group. Compared with the uncertain-compliance group (<60% compliance with ACSM recommendations), the high-compliance group showed significant improvement only in stiffness measurements. The effects of exercise in KOA may depend on the type of exercise and the outcome of interest. Whether the exercise regimen recommended by ACSM is effective in KOA patients needs further study. Therefore, we suggest that future multidimensional exploration and research on appropriate exercise regimens for KOA patients is required.

## Data Availability

The raw data supporting the conclusions of this article will be made available by the authors, without undue reservation.
